# *In Vivo* Biostimulatory Efficacy of Ascorbic Acid-Loaded Poly(lactic-*co*-glycolic Acid) Nanoparticles Hydrogel for Dermal Remodeling

**DOI:** 10.1021/acsomega.5c01910

**Published:** 2025-09-11

**Authors:** Anna Raphaella Autran Colaço, Priscila de Souza Furtado, Daniel Figueiredo Vanzan, Nicole Serqueira da Silva, Flávia Almada do Carmo, Lucio Mendes Cabral, Alice Simon, Jônatas Caldeira Esteves, Plínio Cunha Sathler

**Affiliations:** † Departamento de Análises Clínicas e Toxicológicas, Faculdade de Farmácia, 28125 Universidade Federal do Rio de Janeiro, Ilha do Fundão, CEP 21941-902 Rio de Janeiro, Rio de Janeiro, Brazil; ‡ Departamento de Fármacos e Medicamentos, Faculdade de Farmácia, Universidade Federal do Rio de Janeiro, Ilha do Fundão, CEP 21941-902 Rio de Janeiro, Rio de Janeiro, Brazil; § Departamento de Clínica Odontológica, Faculdade de Odontologia, Universidade Federal do Rio de Janeiro, Ilha do Fundão, CEP 21941-902 Rio de Janeiro, Rio de Janeiro, Brazil

## Abstract

Synthetic, biocompatible, and biodegradable polymers such as poly-l-lactic acid (PLLA) and polycaprolactone (PCL) are used in the form of microparticles (25–200 μm) dispersed in injectable hydrogels for collagen biostimulation through mechanical stimuli and subclinical inflammatory responses. However, their clinical application is limited by adverse effects, including nodule formation and vascular occlusion. As an alternative, nanoparticles based on poly­(lactic-*co*-glycolic acid) (PLGA), a synthetic, biocompatible, and biodegradable copolymer, loaded with ascorbic acid (AA), a potent antioxidant with pro-collagenic activity, may provide enhanced dermal retention, reduced macrophage recognition, and minimized adverse effects. This study developed AA-loaded PLGA nanoparticles incorporated into a hydrogel and evaluated their potential for *in vivo* dermal biostimulation. Nanoparticles were prepared by nanoprecipitation and characterized by dynamic light scattering (DLS) to determine average diameter and polydispersity index (PDI), zeta potential to assess surface charge, and transmission electron microscopy (TEM) to examine morphology. AA encapsulation efficiency was assessed spectrophotometrically. Cytotoxicity was evaluated by the MTT assay using epithelial, macrophage, and fibroblast cells. The hydrogel was evaluated for stability and spreadability. The biostimulatory potential of the formulations was assessed *in vivo* by measuring hydroxyproline levels, collagen production, and dermal remodeling. The nanoparticles exhibited an average diameter below 300 nm, a moderately monodisperse distribution (PDI <0.2), spherical morphology, and high AA encapsulation efficiency (>94%). Biocompatibility was confirmed in epithelial and fibroblast cells (viability >70%), with cytotoxicity observed only in macrophages at higher concentrations (<70%). *In vivo*, AA-loaded PLGA nanoparticles (AANps) induced a 28% increase in collagen production at 30 days, although this effect declined by 60 days. Collagen fibers remained denser and more organized, suggesting ongoing tissue remodeling. Prospectively, after one month, the AANps hydrogel developed in this study could promote a more pronounced collagen increase compared to commercial products such as calcium hydroxylapatite (CaHA) microparticles and PLLA microparticles. These findings suggest that the AA-loaded PLGA nanoparticle hydrogel is a promising strategy for dermal remodeling.

## Graphical abstract

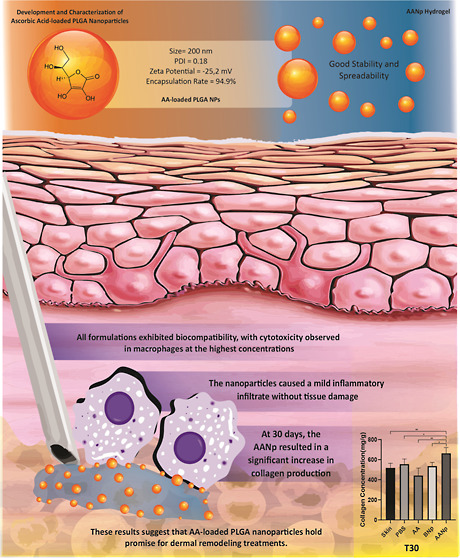

## Introduction

Skin aging, caused by external factors such as smoking and excessive exposure to UVA and UVB radiation, or by internal factors such as infections, acne, and physiological or hormonal conditions, has significantly driven the search for biomaterials with collagen biostimulatory properties.^1,2^ In recent years, the use of synthetic, biocompatible, and biodegradable polymers such as poly-l-lactic acid (PLLA) and polycaprolactone (PCL) in the form of microparticles (25–200 μm) dispersed in injectable hydrogels for collagen biostimulation has been investigated. Their effect occurs through mechanical stimuli to the tissue and the induction of a subclinical inflammatory response,^3^ which promotes macrophage activation and the release of cytokines and growth factors, as well as stimulating collagen and fibronectin synthesis by skin fibroblasts, culminating in dermal remodeling.^4–6^

Despite promising results, microparticles have limitations, such as contraindications for use in specific facial areas due to the risk of nodules, granulomas, and obstruction of blood flow.^7,8^ In this context, nanotechnology has emerged as a promising alternative. One of the key advantages of using nanoparticles as collagen biostimulators is the significant increase in surface area relative to particle volume. This increased surface area allows for enhanced interaction with target tissue cells, particularly fibroblasts and macrophages, thereby amplifying the biostimulatory effect. The larger surface area also promotes the controlled and sustained release of bioactive signals, such as cytokines and growth factors, which can more effectively stimulate collagen production, leading to more robust and lasting tissue regeneration.^9^ Additionally, nanoparticles offer new therapeutic possibilities through the development of polymeric nanosystems capable of carrying bioactive molecules with biostimulatory potential, while providing improved penetrability properties, further enhancing their effectiveness in tissue repair and regeneration.^7,10^

Among the materials used in nanoparticle formulations, poly­(lactic-*co*-glycolic acid) (PLGA), a synthetic, biocompatible, and biodegradable copolymer, has shown good results in promoting collagen synthesis and serving as a carrier for various bioactive compounds.^11^ Ascorbic acid (AA), known for its antioxidant properties and its role in collagen synthesis and stabilization of pro-collagen mRNA, is a key agent in the prevention and control of skin aging and degradation.^12–14^ AA has been shown to stimulate fibroblast proliferation and activity, leading to increased collagen production and improved organization and thickness of collagen fibers.^13^

Given the need to develop and improve new biostimulating products and considering the advantageous properties of both PLGA and AA, the formulation of a biostimulating hydrogel containing PLGA polymeric nanoparticles loaded with AA emerges as a promising approach. This material may have the potential to offer biostimulation and dermal remodeling with fewer complications and restrictions compared to microparticles. To the best of our knowledge, the effect of the combination of the hydrogel with these nanoparticles on the collaged production in skin was not tested before. Thus, the overall aim of this study was to develop, characterize, and evaluate the biostimulating potential of a hydrogel containing AA loaded PLGA nanoparticles as an innovative therapeutic approach for dermal remodeling.

## Material and Methods

### Preparation of the Polymeric Nanoparticles

The production of PLGA nanoparticles (BNp—blank nanoparticles) and AA-loaded PLGA nanoparticles (AANp) was carried out using the nanoprecipitation method. For the BNp, the organic phase was initially prepared by dissolving 37.5 mg of PLGA 50:50 (RESOMER RG 504H, Evonik, USA) in 2.5 mL of acetone (Tedia, Brazil) under moderate agitation (Fisatom, Biovera, Rio de Janeiro, Brazil), resulting in a 15 mg/mL copolymer solution. The aqueous phase was prepared by dispersing 37.5 mg of the surfactant Poloxamer 188 (Sigma-Aldrich, St. Louis, USA) in 7.5 mL of distilled water under moderate agitation. The organic phase was then added dropwise to the aqueous phase under continuous stirring at 350 rpm, with dripping into the center of the aqueous vortex. Stirring was maintained for 5 min, followed by solvent removal in a rotary evaporator (Biovera, Rio de Janeiro, Brazil) for 40 min. The AANp formulation was prepared using a similar procedure, with the addition of 1.3 mg of AA (Sigma-Aldrich, St. Louis, USA) to the organic phase.^15,16^

### Characterization of the Polymeric Nanoparticles

The nanoparticle size and polydispersity index (PDI) were determined using Dynamic Light Scattering (DLS) on a Zeta Sizer S90 (Malvern Instruments, Malvern, UK). The measurements were conducted with a polystyrene cuvette of the dip cell type, with a 90° detection angle, a refractive index of 1.33, temperature control maintained at 25 °C, and stirring speed of 350 rpm. The analyses were performed in triplicate, with each measurement representing the average of ten evaluation cycles.^17^

The zeta potential (ζ) was determined using the Phase Analysis Light Scattering technique with the NanoBrook ZetaPALS Potential Analyzer (Brookhaven Instruments Co., Holtsville, NY, USA). Prior to the analysis, pH measurements were taken for the samples, which were then diluted in a 1:2 ratio with distilled water. The assessments were conducted in triplicate, with ten measurements taken for each sample to calculate the average value.^17^

The morphology of the nanoparticles was assessed using transmission electron microscopy (TEM). For this evaluation, 10 μL of the nanoparticle dispersion was applied to 400-mesh copper grids coated with a Formvar film and carbon. The grids were then dried with filter paper, and 10 μL of 5% uranyl acetate was added for 2 min. Finally, the Np were stored in a desiccator with silica overnight before being observed and analyzed using a Tecnai Spirit 120 kV microscope.^17^

### Quantification of Ascorbic Acid and Encapsulation Efficiency

The AA quantification was performed using a metachromatic reaction with spectrophotometric measurement (λ = 546 nm) according to the previously described method with minor modifications.^18^ This reaction involved the reduction of iron­(III) to iron­(II), followed by the complexation of iron­(II) using 1,10-phenanthroline (Phen) solution (Sigma-Aldrich, St. Louis, USA).

The reagent solutions were prepared as described below. The Phen stock solution (0.01 M) was prepared by dissolving 0.46 g of 1,10-phenanthroline monohydrate (Sigma-Aldrich, St. Louis, USA) in 250 mL of distilled water. The 0.003 M iron solution was prepared by dissolving 0.54 g of iron chloride hexahydrate (Cl_3_FeH_12_O_6_, Sigma-Aldrich, St. Louis, USA) in 1000 mL of distilled water. The copper solution (pH 6.0) was prepared by dissolving 20 mg of copper sulfate (CuSO_4_, Sigma-Aldrich, St. Louis, USA) in 200 mL of 1 M sodium acetate solution, followed by the addition of 7 mL of acetic acid (CH_3_COOH, Sigma-Aldrich, St. Louis, USA) and completing with distilled water to 1000 mL. The 0.05 M ethylenediaminetetraacetic acid (EDTA) solution was prepared by dissolving 9.25 g of EDTA sodium salt (C_10_H_14_N_2_Na_2_O_8_, Sigma-Aldrich, St. Louis, USA) in 500 mL of distilled water.

For quantification assay, the final standard and sample solutions were prepared in a 25 mL volumetric flask, and after standard or sample addition, the reagent solutions were added in sequence (2.5 mL of 0.003 M iron solution, 2.5 mL of copper solution and 2.5 mL of 0.01 M Phen solution). After 1 min of mechanical stirring (75XA FISATOM) at room temperature (24 °C), 0.5 mL of 0.05 M EDTA solution was added, the volumetric flask was filled with distilled water and stirred. A blank solution was prepared without addition of standard or sample (just reagents). Next, the volume of 200 μL of standard/samples/blank solutions were singly transferred to a 96-well plate for absorbance measurement at 546 nm using a spectrophotometer (Kasuaki DR-200BS-NM). The linearity curve of AA was constructed concentrations ranging from 1 to 40 μg/mL and the detection (LOD) and quantification (LOQ) limits were calculated using the equations: LOD = (3.3 × σ)/IC and LOQ = (10 × σ)/IC, where IC is the slope of the linearity curve and σ is the standard deviation of the intercept.

The encapsulation efficiency percentage (%EE) of AA was determined by an indirect method, involving ultracentrifugation to quantify the free AA in the supernatant, which represents the fraction not encapsulated in the Nps. For %EE evaluation, the Nps were separated from the aqueous medium by ultracentrifugation at 48,000*g* using a HITACHI High-Speed Refrigerated Centrifuge CR22N for 30 min. The %EE was calculated using the equation: %EE = [(AA_total_ – AA_free_)/AA_total_] × 100, where AA_total_ is the amount of AA used in Np preparation (mg) and AA_free_ is the free amount of AA quantified in the wash solution/supernatant (mg).^19^

### Preparation of Hydrogel Containing Polymeric Nanoparticles

To prepare the hydrogel, 0.0175 g of methylparaben (Nipagin, ISOFAR, Rio de Janeiro, Brazil) was dissolved in 8.75 mL of distilled water with manual stirring. A second solution was prepared by dissolving of 0.33 g of carboxymethylcellulose (CMC) (Neon Quimica, São Paulo, Brazil) in 8.75 mL of distilled water, at room temperature. Following this, the preservative solution was gradually incorporated into the CMC solution using a glass rod for mixing. After achieving a homogeneous mixture, a volume of 17.5 mL of hydrogel at a concentration of 1.88% (w/v) CMC was obtained.^18,20^ Subsequently, 7.5 mL of the AANp solution was incorporated into the hydrogel, and after manual homogenization, a final volume of 25 mL of hydrogel containing AANp was obtained. The same procedure was used for preparing the hydrogel containing BNPs. Finally, the appearance, stability and spreadability of the hydrogel formulations were analyzed.

The appearance evaluation of the hydrogel formulations was conducted macroscopically, focusing on color, physical aspects, and the state of the hydrogel over a 30 day period at room temperature (approximately 30 °C).^20^ The spreading capacity of the hydrogel formulations was assessed after exposure for 1 min to predetermined weights. On millimeter paper, a glass slide was outlined using a ruler, with diagonals drawn from corner to corner to form an “*X*”. The slide was then placed over the outline, and a projection pen was used to mark a point at the intersection of the lines. Twenty-five mg of the sample was deposited on this point. A new glass slide was weighed and recorded. The slide with the sample was positioned over the drawing on the millimeter paper, and a weighed slide was placed on top for 1 min, using a stopwatch. Afterward, the diameter of the formed circle was recorded. A 2 g weight was added on top the weighed glass slide, and another minute was timed. This process continued until the final weight was reached. After obtaining the final diameter at each stage, spreadability was calculated using the equation: *Ei*= (*d* ^2^ × π)/4, where *Ei* = spreadability of the sample for a given weight (cm^2^); *d* = diameter (cm).^50^

### Cytotoxicity Assay

The epithelial (Vero/code 0245), macrophage (J774A.1/code 0121), and fibroblast (HFF-1/code 0275) cell lines were obtained from the Rio de Janeiro Cell Bank (Rio de Janeiro, Brazil). Vero cells were cultured in Dulbecco’s Modified Eagle’s Medium (DMEM; Sigma-Aldrich, code D6046, Brazil) low glucose, supplemented with 10% fetal bovine serum (FBS; Sigma-Aldrich, code F2442, Brazil) and 1% penicillin/streptomycin antibiotic (10,000 U/mL and 10,000 μg/mL, respectively; Nova Biotecnologia, code BR30110-01, Brazil). Fibroblasts and macrophages were cultured under the same conditions, but using high glucose DMEM (Sigma-Aldrich, code D0819, Brazil) with 15% FBS. All cultures were maintained in an incubator at 37 °C with 5% CO_2_ and 95% atmospheric air, subcultured every 72 h with fresh medium until the cells reached semiconfluence (≥80%).

Two methods were used for cell passaging. For Vero and HFF-1 cells, 1 to 2 mL of a 0.25% trypsin–EDTA solution (Sigma-Aldrich, code T3924, Brazil) was applied for 7 to 10 min, depending on the confluence of the culture flask (25 or 75 cm^2^, Kasvi, RJ, Brazil). Trypsin solution was neutralized by adding twice the volume of culture medium (DMEM −10% or 20% FBS). For J774A.1 cells, a cell scraper was used to detach the cells from the flasks along with 2 mL of DMEM-15% FBS. An additional 2 mL of medium was then added to the flask to ensure the recovery of all remaining cells. All cultures were subjected to centrifugation at 2500 rpm for 7 to 10 min. The resulting supernatant was discarded, and the cell pellet was resuspended in complete medium.

Vero (passage 30), J774A.1 (passage 5), and HFF-1 cells (passage 28) were seeded in 96-well plates at densities of 1 × 10^4^, 2 × 10^4^, and 3 × 10^4^ cells/well, respectively, using 200 μL of DMEM-10% FBS for Vero and DMEM-15% FBS for J774A.1 and HFF-1. The cells were incubated at 37 °C with 5% CO_2_ until they reached 80% confluence. The growth medium was then replaced with 200 μL of test samples (Poloxamer 188, AA, BNp, and AANp) in supplemented medium, with concentrations ranging from 6.25 to 100 μg/mL. The plates containing AA and AANp were protected from light. Supplemented medium was used as a negative control and pure DMSO as a positive control. The cells were incubated at 37 °C with 5% CO_2_ for 24 h, all analyses were performed in quadruplicate. After the incubation period, the medium containing test samples was removed, and each well received 100 μL of MTT (0.5 mg/mL in PBS, pH 7.4). The plates were protected from light, and the cells were incubated at 37 °C with 5% CO_2_ and 95% atmospheric air for 3 h. Subsequently, the formazan crystals were dissolved in 100 μL of DMSO. Absorbance was measured at 562 nm using a Kasuaki DR-200BS-NM spectrophotometer. To determine cell viability, the results of the samples were compared to untreated cells (negative control), which were taken as 100% viability.

### Ethical Aspects in Animal Experimentation

For the conduct of experiments involving animals, the project protocol was submitted to and approved by the Animal Ethics Committee (CEUA) of the Center for Health Sciences at the Federal University of Rio de Janeiro, under protocol number 035/22. All animal experiments followed to ARRIVE guidelines and were conducted in accordance with the UK’s Animal (Scientific Procedures) Act 1986 and its associated guidelines, the European Communities Council Directive of November 24, 1986 (86/609/EEC), and the Guide for the Care and Use of Laboratory Animals.

In this study, male Wistar rats (*Rattus norvegicus albinus*), aged 8 to 10 weeks, were obtained from the Animal Facility of the Faculty of Pharmacy at the Federal University of Rio de Janeiro. The choice for the use of male rats was due to avoid the interference of hormonal rates observed in female rats. During both the growth and experimental periods, the animals were maintained under controlled laboratory conditions, including adequate hygiene, a room temperature of 24 ± 2 °C with a 12 h light/dark cycle, and each cage housed a maximum of four animals. Throughout the experiments, the rats had free access to standard commercial food and water.

### Assessment of Collagen Production and Dermal Remodeling *In Vivo*

The animals were divided into five experimental groups, and samples were collected at the time intervals of 0, 15, 30, and 60 days, with four animals at time 0 and eight in each subsequent time point: (i) negative control: 1% PBS solution (0.2 mL; retro-injection 2 cm into the subcutaneous fat tissue); (ii) AANp: hydrogel containing AANp (0.2 mL retro-injection of 2 cm into the subcutaneous fat tissue—AA = 0.0104 mg, PLGA = 0.3 mg, CMC = 2.64 mg); (iii) BNp: Hydrogel containing PLGA Np (0.2 mL retro-injection of 2 cm into the subcutaneous fat tissue—PLGA = 0.3 mg, CMC = 2.64 mg); (iv) AA: application of AA (0.2 mL retro-injection of 2 cm into the subcutaneous fat tissue—AA = 0.0104 mg); (v) baseline control: no pharmacological intervention was performed.

Initially, the rats were anesthetized by intramuscular injection with ketamine hydrochloride (100 mg/kg body weight) and xylazine hydrochloride (25 mg/kg body weight) solutions. Subsequently, manual shaving, antisepsis with 70% ethanol, and dorsal marking for injection orientation were performed. The formulations were administered as 0.2 mL subcutaneous injections in a linear track of 2 cm, positioned 1 cm away from the spine on both sides. On the left hemidorsum, the formulation (hydrogel with AANp; hydrogel with BNp) was always administered, while on the right side, the 1% PBS solution was injected. The animals were monitored for 15, 30, and 60 days, and euthanized after these periods with an overdose of anesthetic.

The baseline control animals did not receive any formulation or dorsal puncture. They were euthanized with the association of the isoflurane and a cardiac injection of potassium chloride (3 M—Sigma-Aldrich, St. Louis, USA). Subsequently, a 10 × 8 mm skin and subcutaneous tissue samples from the animals were collected and divided into two similarly sized segments, approximately 2 cm^2^ each. One segment was fixed in 4% paraformaldehyde (Synth, Rio Grande do Sul, Brazil) solution in PBS pH 7.4 for 48 h and then processed for routine histological analysis. The fixed tissues were sectioned into 4 μm thick slices, which were stained with Hematoxylin & Eosin (H&E—Sigma-Aldrich, St. Louis, USA) and Picrosirius Red (Sigma-Aldrich, St. Louis, USA). These histological sections were analyzed for histomorphological and histomorphometric evaluation to assess collagen fiber formation within the tissue (Ray & Ta, 2020) with the aid of an optical microscopy AXIO Zoom.V16 (EMS3/SyCoP3, Zeiss).

### Hydroxyproline/Collagen Determination

The hydroxyproline was determinated through a colorimetric redox assay, in which the complexation of hydroxyproline with chloramine T (Sigma-Aldrich, cat. no. 857319) and the oxidation of this complex by *p*-dimethylaminobenzaldehyde (Sigma-Aldrich, cat. no. 109762) produced a reddish coloration (Cissell et al., 2017). For this assay, fragments of fresh skin from the treated area were weighed and preprocessed. The preprocessing commenced with a dehydration step, involving immersion of the tissue in acetone until complete dehydration was achieved. In the subsequent step, the sample was subjected to maceration, and 20 mg of tissue was weighed into test tubes. Following this, the samples were processed via hydrolysis to break peptide bonds using 1 mL of 6 N hydrochloric acid (HCl) under heating at 107 °C in a dry bath within a fume hood, until complete evaporation of the liquid occurred. After hydrolysis, the material was suspended in 1 mL of citric acid buffer at pH 6.0, transferred to 1.5 mL Eppendorf tubes, and centrifuged at 8000 rpm for 10 min. The final processing step involved a 10-fold dilution (1:10) with citric acid buffer at pH 6.0.

To initiate the hydroxyproline determination assay, 100 μL of sample or hydroxyproline standard was transferred to test tubes, and 625 μL of chloramine T solution was added to each tube containing the samples. The solution was then homogenized and allowed to stand for 20 min. The next step, 625 μL of Ehrlich’s reagent was added, homogenized, and heated at 65 °C in a water bath for 20 min. After this time, the samples and standard were cooled in ice water to stop the reaction, and 200 μL of the resulting material was transferred to a 96-well plate for absorbance measurement at 562 nm using a Kasuaki DR-200BS-NM spectrophotometer.

The amount of hydroxyproline in the tissue was determined using a calibration curve based on an analytical standard of hydroxyproline (Sigma-Aldrich, cat. no. 41875). The calibration curve was established between the concentration range of 12.5–200 μg/mL. The linearity of the curve was confirmed by assessing the correlation coefficient, limit of detection, limit of quantification, and slope (Supporting Information: Figure S1).

### Determination of Sample Selection for Histomorphological Analysis

For histomorphological analysis, the three most central histological sections were examined. These sections were stained with Hematoxylin and Eosin (H&E) and Picrosirius Red and analyzed using light microscopy to qualitatively assess the tissue response to the injections of formulations and the control group. A region of interest (ROI) of 2000 × 1000 μm located 10 mm from the dorsal marking on the animal, was identified using the linear measurement tool in ImageJ software (NIH, Bethesda, MD, USA). The histomorphological characteristics of the subcutaneous tissue, including collagen fiber density and thickness, cell types, inflammatory infiltrate, and skin thickness, were analyzed.

The intensity of the inflammatory response was assessed using a semiquantitative scoring system in the HE-stained slides: 0: absence of inflammatory cells; 1: mild inflammation (few inflammatory cells); 2: moderate inflammation (many inflammatory cells); 3: severe inflammation (prevalence of inflammatory cells).^21^ To assess collagen fiber maturation, the picrosirius-stained slides were photographed under dark field and nonpolarized light conditions. Under these conditions, reticulated and mature collagen fibers appear red, while immature collagen is displayed in shades of green and orange.^22^

### Statistical Analysis

The data were subjected to statistical analysis for intergroup comparisons. Initially, the normality of the data was tested using the Kolmogorov–Smirnov and Shapiro–Wilk tests. Given that data were normally distributed, one-way ANOVA followed by Tukey’s post hoc test was used for group comparisons. Statistical tests were performed with a confidence level of 95% using GraphPad Prism 8.0 software (San Diego, CA, USA). Regarding the cytotoxicity analysis, the results were analyzed using one-way analysis of variance (ANOVA) followed by the Dunnett’s multiple comparison test. The p value ≤0.05 was considered statistically significant. Data were expressed as mean ± standard deviation (SD).

## Results and Discussion

### Preparation and Characterization of Polymer Nanoparticles

The nanoprecipitation method employed involves selecting a solvent, which is both miscible with water and capable of solubilizing the drug and polymer. When this solvent is introduced into an aqueous solution containing a surfactant, the diffusion between the two phases promotes the precipitation of NPs, resulting in their dispersion. Acetone is used as the organic solvent due to its water-miscibility and its ability to effectively solubilize AA. In this process, the organic phase consists of acetone, PLGA polymer, and AA, while the aqueous phase comprises Poloxamer 188 surfactant dissolved in water. (Table 1).

**Table 1:** Characterization of the Nps Formulations Tested in This Study

Type of Np		BNp	AANp
Composition	PLGA (mg)	37.5	37.5
	acetone (mL)	2.5	2.5
	poloxamer 188 (mg)	37.5	37.5
	water (mL)	7.5	7.5
	AA (mg)		1.3
size (nm)		211.25 ± 3.5	200.07 ± 11.30
PDI		0.192 ± 0.01	0.183 ± 0.01
pH		4.77 ± 5.06	4.11 ± 3.55
zeta potential (mV)		–37.8 ± 3.5	–25.2 ± 0.9
encapsulation efficiency (%)			94.87 ± 0.38
spreadability (cm^2^)	slide (5.09 g)	3.14	4.91
	slide +2 g	4.15	5.31
	slide +2 g + 2 g	4.52	5.72
	slide +2 g + 2 g + 5 g	4.52	6.16

### Determination of the Average Diameter and Polydispersity Index of the Polymer Nanoparticles

The average diameter and PDI of the Nps were analyzed using DLS techniques. The BNp and AANp exhibited size values consistent with the nanometric classification, measuring 211.23 ± 3.50 nm (PDI 0.192 ± 0.008) and 200.06 ± 11.34 nm (PDI 0.183 ± 0.010) for BNp and AANp, respectively. The PDI, measured using the same equipment and methodology, is a parameter that indicates the relative polydispersity of the Np sizes; in this context, values closer to 0 suggest more homogeneous, or monodisperse, systems. According to the literature, the prepared formulations exhibited the expected profile, being within the nanometric scale and moderately polydisperse^23,24^ (Table 1).

### Zeta Potential Determination of Polymer Nanoparticles

Determining and evaluating the electrical potential is crucial, because the surface charge of the Nps can provide an estimate of the colloidal stability of the formulations. Initially, a slightly acidic pH, which is close to the skin’s pH, was achieved, potentially enhancing application comfort. In this context, the measured pH may have been influenced by both the polymer (PLGA) and the AA present in the formulations (Table 1).

In this study, the zeta potential of AANp was −25.2 ± 0.9 mV, while that of BNp was −37.8 ± 3.5 mV, showing a strong correlation with the pH of the respective dispersions: 4.11 ± 3.55 for AANp and 4.77 ± 5.06 for BNp. This electronegative character can be attributed to the presence of the PLGA copolymer, whose monomers, lactic acid (p*K*_a_ ≈ 3.86) and glycolic acid (p*K*_a_ ≈ 3.83), contain terminal carboxylic acid groups.^25^ The proximity between the dispersion pH values and the p*K*_a_ of these monomers suggests that approximately 50% of the carboxylic acid groups are ionized, contributing to the observed negative surface charge.^26^

Traditionally, a zeta potential of at least ±30 mV is associated with sufficient electrostatic repulsion to maintain colloidal stability. Accordingly, BNp meets this criterion. However, AANp, despite exhibiting a lower zeta potential (−25.2 mV), remained stable for 24 h with no visible signs of precipitation or aggregation. This observation suggests that mechanisms beyond electrostatic stabilization may be involved.

Indeed, in systems stabilized with nonionic surfactants or polymeric chains—such as those possibly present in AANp—steric stabilization plays a significant role. In this mechanism, nonionic or polymeric moieties adsorbed onto the nanoparticle surface form a hydrated, flexible layer. When particles approach one another, the overlapping of these surface-bound chains leads to entropic and osmotic repulsion, creating a physical barrier that inhibits aggregation.^27^ Thus, the colloidal stability of AANp may be attributed not only to its moderate surface charge but also to steric effects provided by nonionic stabilizing components. This dual mechanism is consistent with previous studies reporting stable colloidal systems exhibiting low zeta potential but high steric protection.^28^

### AA Encapsulation Efficiency

The samples demonstrated a high encapsulation rate of AA, with over 94% of AA encapsulated within the nanosystem, indicating that the nanoparticle was efficiently loaded with AA. Previous studies reported high encapsulation efficiencies for Nps of 70% to 85%. The values obtained for development of AANp were even higher than those reported in these studies, highlighting the success of the encapsulation method used in this work^29–32^ (Table 1).

### Study of Nanoparticle Morphology by Transmission Electron Microscopy

TEM analysis confirmed the findings from DLS regarding nanoparticle size, confirming that the nanoparticles are indeed within the nanometric scale. The nanoscale size of the particles improves the encapsulation of the molecules^33^ and could be the reason for the good outcomes in the encapsulation of the AA presented in this study. Additionally, TEM observations revealed that the nanoparticles were spherical with smooth edges, and that the incorporation of AA did not alter their morphology (Figure 1). The maintenance of the morphology of the NPs after the AA incorporation may impact the controlled release of the AA after their use, since this effect is important in the maintenance of the molecule encapsulated into the NPs for longer periods.^34^ Furthermore, the rounded shape and smooth edges improves the dispersion of the NPs into the tissues^35^ (Table 1).

**Figure 1:**
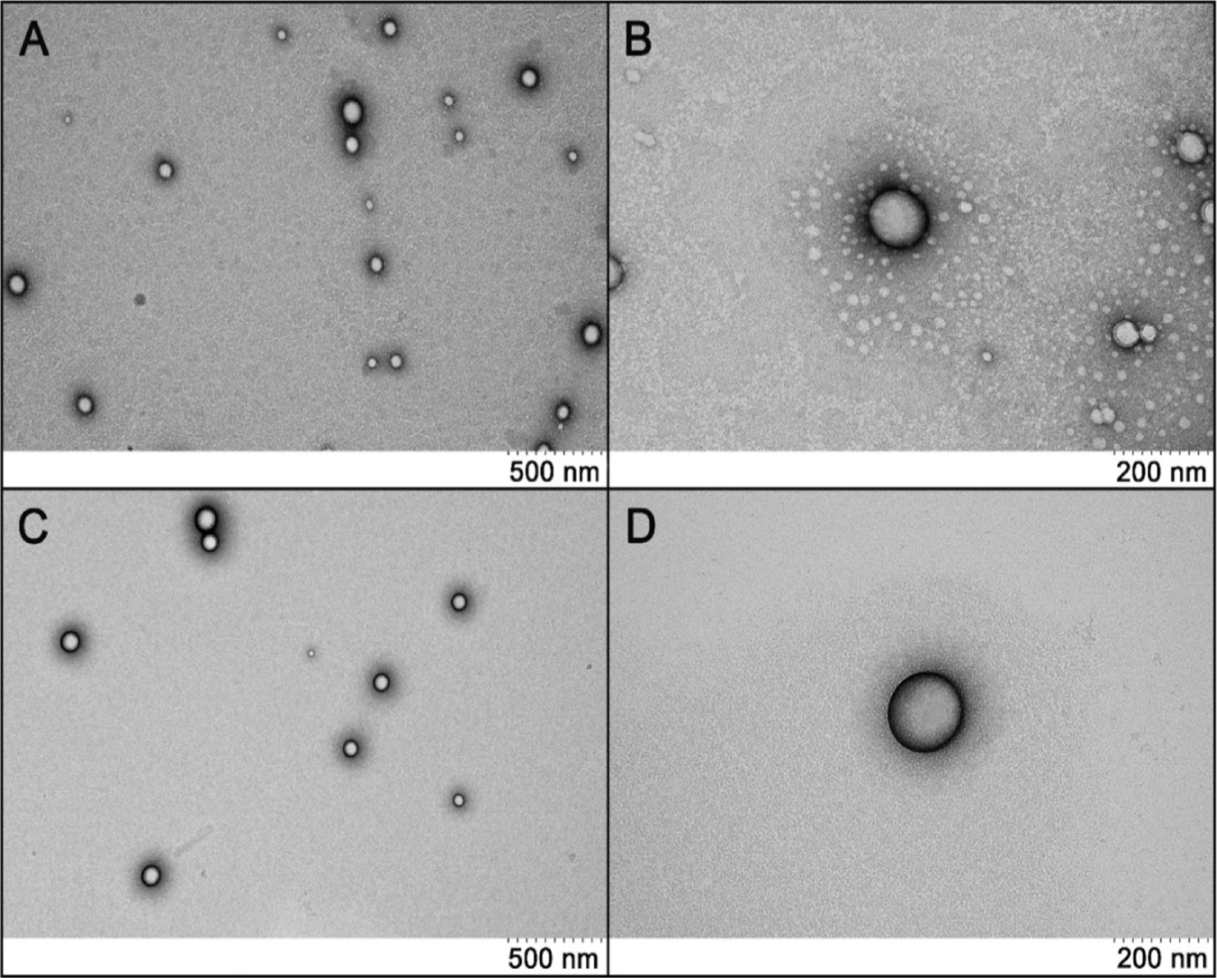
Representative images of the nanoparticle morphology analysis using transmission electron microscopy: (A) BNp 20x; (B) BNp 50x; (C) AANp 20x; (D) AANp 50x. These images show that the particles are on a nanometric scale, have a rounded shape, and do not exhibit significant morphological differences resulting from AA incorporation.

### Hydrogel Analysis

Spreadability of a substance refers to its capacity to uniformly cover a surface, a property influenced by factors such as molecular weight, molecular interactions, and viscosity. In formulations, this dynamic becomes more intricate due to the interplay of interactions among various components.^36^ The spreadability study aimed to assess whether the hydrogel composed of CMC influenced the spreadability of the proposed formulations. Both hydrogels exhibited a semisolid, lightweight, and opaque physical appearance (Supporting Information: Figure S2). No macroscopic changes were observed over 30 days when maintained at room temperature close to 30 °C. The spreadability was assessed with gels containing both BNp and AANp. It was noted that the hydrogel containing the nanosystem with AA showed better spreadability in the test, but without significant difference (Table 1).

### *In Vitro* Cytotoxicity Study

To investigate the biocompatibility of the BNp and AANp formulations, we assessed their interactions in epithelial cells (Vero), Balb/C mouse macrophages (J774A.1), and human skin fibroblasts (HFF-1) at concentrations ranging from 6.25 to 100 μg/mL after 24 h of incubation using the MTT reduction assay (Figure 2). Additionally, we evaluated the cytotoxicity of the individual components, Poloxamer 188 and AA, under the same experimental conditions (Supporting Information: Figure S3).

**Figure 2:**
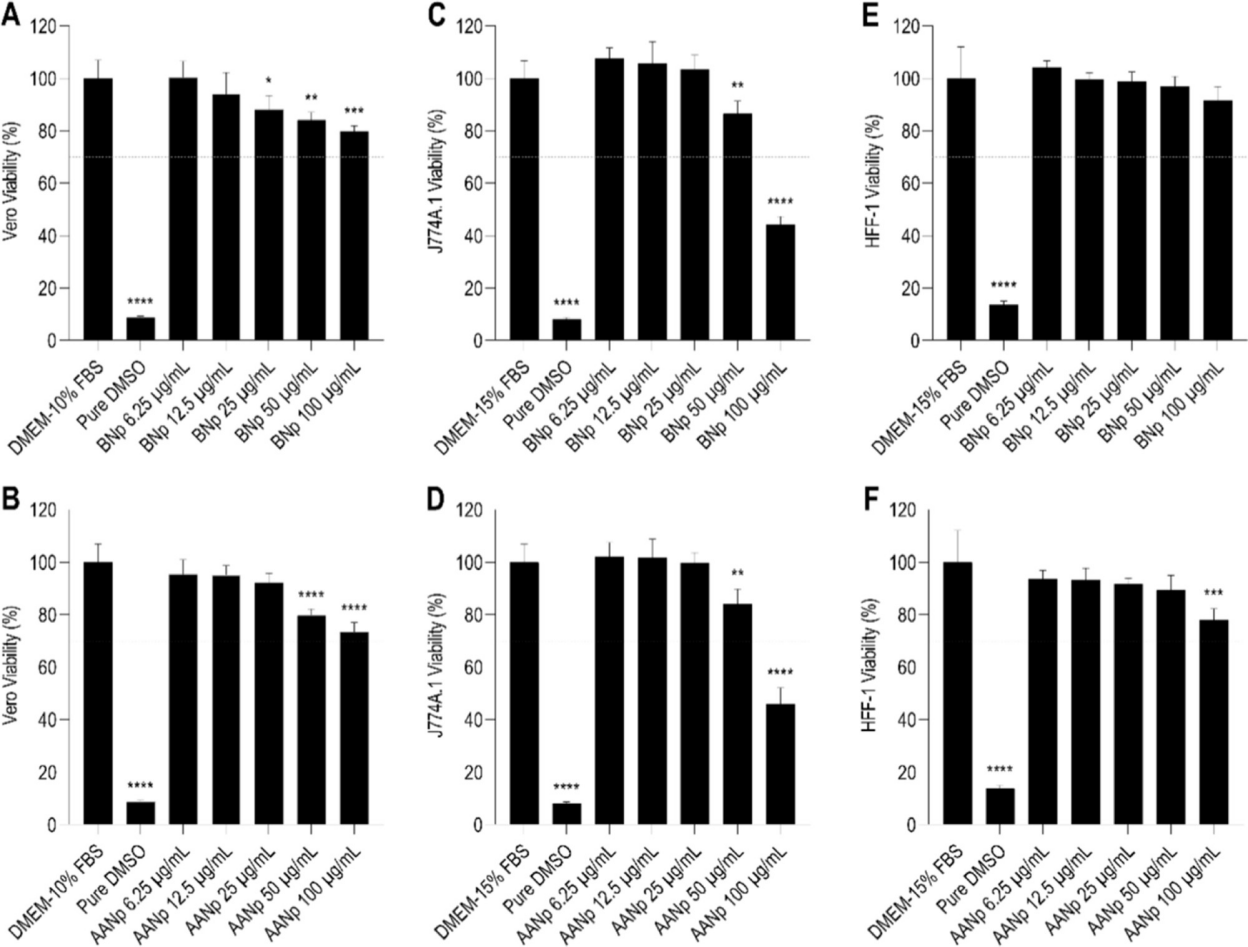
*In vitro* assessment of the cytotoxic effect of BNp and AANp on Vero (A,B), J774A.1 (C,D), and HFF-1 (E,F) cells at concentrations ranging from 6.25 to 100 μg/mL. DMEM-10% FBS and DMEM-15% FBS were used as negative controls, and pure DMSO as the positive control. Results are expressed as mean ± standard deviation (SD); *n* = 4 (*p < 0.05; **p < 0.005; ***p < 0.0005; ****p < 0.0001).

No cytotoxic effects were observed in Vero cells exposed to the BNp and AANp formulations at concentrations of up to 100 μg/mL, with viability values recorded at 79.82 ± 2.11% (*p* < 0.0005) and 73.42 ± 3.58% (*p* < 0.0001), respectively, compared to the negative control (100.00 ± 6.95%) (Figures 2A,B). Additionally, free AA exhibited no cytotoxic potential in Vero cells at the same concentration, with a viability of 74.94 ± 4.80% (*p* < 0.0001) (Supporting Information: Figure S3), while the surfactant Poloxamer 188 also demonstrated no cytotoxic effects at 100 μg/mL, with a viability of 77.50 ± 8.00% (*p* < 0.005) (Supporting Information: Figure S3A). These results indicate that neither the formulations nor their individual components induced significant reductions in Vero cell viability, even at the highest tested concentrations. This is consistent with ISO 10993-5:2009 standards, which state that a reduction in cell viability greater than 30% is considered a cytotoxic effect.

J774A.1 cells exposed to BNp and AANp at concentrations of up to 50 μg/mL maintained cell viabilities of 86.41 ± 4.91% (*p* < 0.0001) (Figure 2C) and 84.10 ± 5.69% (*p* < 0.0001) (Figure 2D), respectively, compared to the negative control (100.00 ± 6.78%). However, when incubated with BNp and AANp at the highest concentration tested (100 μg/mL), cell viability values fell below the 70% threshold (44.26 ± 2.97% and 45.77 ± 6.37%, respectively) (*p* > 0.05; *p* < 0.0005) (Figure 2C,D), indicating a potential cytotoxic effect. For Poloxamer 188 and free AA, no cytotoxic effects were observed in J774A.1 up to 50 μg/mL (96.16 ± 4.87% and 84.73 ± 4.89%, respectively) (*p* > 0.05; *p* < 0.0005), with cytotoxicity noted at 100 μg/mL (35.27 ± 2.92% and 28.81 ± 3.97%, respectively) (*p* < 0.0001) (Supporting Information: Figure S3C,D). These findings corroborate the observations of Ray & Ta (2020), which reported J774A.1 cell viabilities below 70% when exposed to concentrations of 300 and 1000 μg/mL of poly-l-lactic acid nanoparticles.^37^

Similarly, no cytotoxicity was observed when HFF-1 cells were exposed to BNp and AANp at concentrations up to 100 μg/mL (91.65 ± 5.21% and 77.90 ± 4.40%, respectively) (*p* < 0.0005) and 73.42 ± 3.58% (*p* > 0.05; *p* < 0.0005), compared to the negative control (100.00 ± 12.05%) (Figure 3E,F). Likewise, Poloxamer 188 and free AA did not exhibit cytotoxic potential in dermal fibroblasts at concentrations up to 100 μg/mL, with values of 88.15 ± 1.17% (*p* < 0.05) (Supporting Information: Figure S3E) and 78.20 ± 7.23% (*p* < 0.005) (Supporting Information: Figure S3F). To ensure a safe and clinically relevant concentration range, it is advisible to use 50 μg/mL for both nanoparticles, as this concentration helps preserve the integrity of the skin and epithelial tissue.

**Figure 3:**
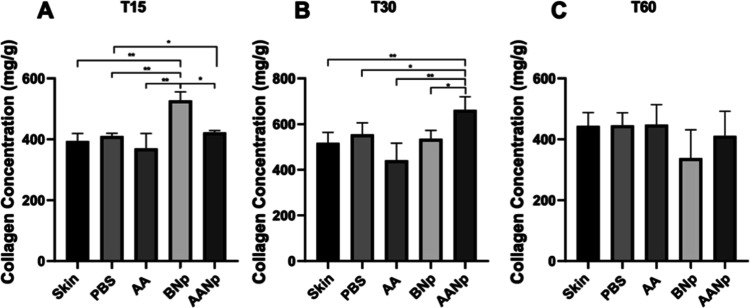
Collagen values, in milligrams per gram of skin at 15 (A), 30 (B), and 60 (C) days in control samples without treatment (skin), treated with PBS, AA, BNp, and AANp (*n* = 4/group). *p < 0.05. **p < 0.01. One-way ANOVA complemented by Tukey’s test.

### Hydroxyproline/Collagen Production

Hydroxyproline is an amino acid present in collagen chains; thus, its quantification allows for the estimation of the collagen percentage in tissue. The mass of hydroxyproline obtained were converted to collagen,^38^ which found that type I collagen, the major constituent of the dermis, contains 13% hydroxyproline. For this study, skin fragments from Wistar rats were used as samples, treated with 1% PBS, AA free, BNp, and AANp. Hydroxyproline levels were measured to assess the impact of these treatments on collagen content in the skin fragments. To investigate this influence, skin fragments from animals were obtained at 15-, 30-, and 60 days posttreatment.

The results obtained from the hydroxyproline/collagen determination of the animal skin fragments are presented in Figure 3.

At 15 days, it was observed that animals receiving BNp showed a significant increase in collagen production compared to the control groups, PBS, and AA (*p* < 0.01), as well as AANp (*p* < 0.05) (Figure 3A). During this initial period, no significant effect of the AANp on collagen production was observed, although statistical significance was noted relative to the PBS group (*p* < 0.05). This finding may be related to the immediate availability of nonencapsulated AA. This could potentially lead to a reduction in the pH of the tissue microenvironment. One study states that this condition could influence the signaling process between fibroblasts and other cell groups by altering cytokine and growth factor activity, thereby suppressing collagen production.^39^ This hypothesis can also be observed in the free ascorbic acid group, where a slight reduction in collagen levels was noted.

Concerning the values obtained 30 days after treatment (Figure 3B), a notable difference was observed compared to 15 day results. The AANp demonstrated a significant increase in collagen production relative to all groups (*p* < 0.05), indicating that these nanosystems, due to controlled release potential, allow AA to remain at the site of action in optimal concentrations without causing an immediate acid environment that could adversely affect collagen production. In contrast, BNp maintained its significant effect on collagen production compared to the control group (*p* < 0.05), although no differences were observed when compared to AANp group (*p* ≥ 0.05).

The results obtained at 15- and 30 days posttreatment suggest that AA exhibits a slow but sustained release profile. This prolonged release may represent a modified-release system capable of extending the duration of the therapeutic effect.^26^ Interestingly, the findings of the present study indicate an advantage over the maintenance of collagen production in microneedling techniques. Indeed, a previous study observed a gradual reduction in dermal thickness and fibroblast count following the peak stimulation (14 days) with gelatin microneedles containing adipose-derived collagen fragments in a murine model.^40^ In contrast, the current system provides continuous stimulation, rather than a single, nonmaintained event.

At the end of 60 days, no significant continuation in collagen production by the nanosystems was observed compared to the controls, indicating a reduction in the efficiency of the nanoparticles over time. This decline is likely due to the metabolic processing of the polymeric structures and ascorbic acid.

The long-term collagen production observed in this study may be attributed to the additional actions of ascorbic acid on the biosynthesis of this molecule,^40,41^ as well as the maintenance of the nanosystem at the application site and the sustained release of AA in situ. This observation suggests that treatment with PLGA nanoparticles containing AA extends the effect of AA on collagen synthesis in the dermis, primarily due to its slow and gradual release. This phenomenon aligns with the known activity of ascorbic acid, which directly influences collagen biosynthesis at various stages, including mRNA transcription responsible for procollagen production and proline hydroxylation to hydroxyproline.^3^

Upon analyzing the collagen profile of the experimental groups after 30 days, it was observed that the AA-containing nanoparticles promoted an approximate 28% increase in collagen production compared to the control group. In this context, based on the study of Goldberg et al. (2013),^42^ PLLA demonstrated a 66.5% increase in this protein within the dermal tissue by the end of the third month posttreatment. In contrast, Yutskovskaya and Kogan (2017)^43^ observed only a 15% increase when using Diluted Calcium Hydroxylapatite (CaHA) in an evaluation conducted in the fourth month. Prospectively, after one month, the AANp developed in this study could potentially achieve a more effective collagen increase than the reported products.^12,44–46^

In addition to the well-known role of AA in collagen production, the hydrogel containing polymeric nanoparticles, when used as an injectable dermal filler, may exert an additional effect through the stimulatory activity of the polymer. This effect occurs due to the increased rigidity of the dermal microenvironment and the interaction between the polymer and the transmembrane integrins of dermal fibroblasts. This process triggers mechanotransduction via the Yes-associated protein/transcriptional coactivator with PDZ-binding motif (YAP/TAZ) pathway, promoting the transcription and gene expression of transforming growth factor beta 1 (TGF-β1) and connective tissue growth factor (CTGF), which in turn stimulate collagen production and the proliferation of dermal fibroblasts.^45,47^ This information supports the activity profile of the BNp observed after 15 days.^44^ Interestingly, this increase in collagen production was not observed in the BNp group 30 days after treatment. The similarity in values between the control and BNp groups at this time suggests a reduction in the mechanotransductive stimulation of the polymer. A previous study reported that the degradation of PLGA nanoparticles becomes noticeable around the seventh day, when pores start to appear in their spherical structure, and by 21 days, the nanoparticles have completely lost their morphology.^48^ These findings corroborate the observed decrease in biostimulatory potential over time, as seen in the comparative graphs.

### Histological Analysis

All animals demonstrated a healing process devoid of evident inflammatory signs, infectious processes, or clinically observable lesions at the injection sites throughout all analysis periods. Microscopic examination at 15 days showed that specimens from all three groups were well-healed, with no histomorphological evidence of tissue damage. A subtle inflammatory infiltrate was observed across all experimental groups. Lymphocytes and macrophages were detected within the dermis, distributed throughout the collagen matrix and subcutaneous fat. Although inflammatory cells were more prevalent in the test groups, none of the specimens exhibited moderate inflammatory infiltrate (Figure 4). During this period, the density and distribution of collagen fibers were similar between the groups. However, in all specimens from the BNp group, the analysis area was predominantly occupied by young collagen fibers, indicating an ongoing neocollagenesis process.

**Figure 4:**
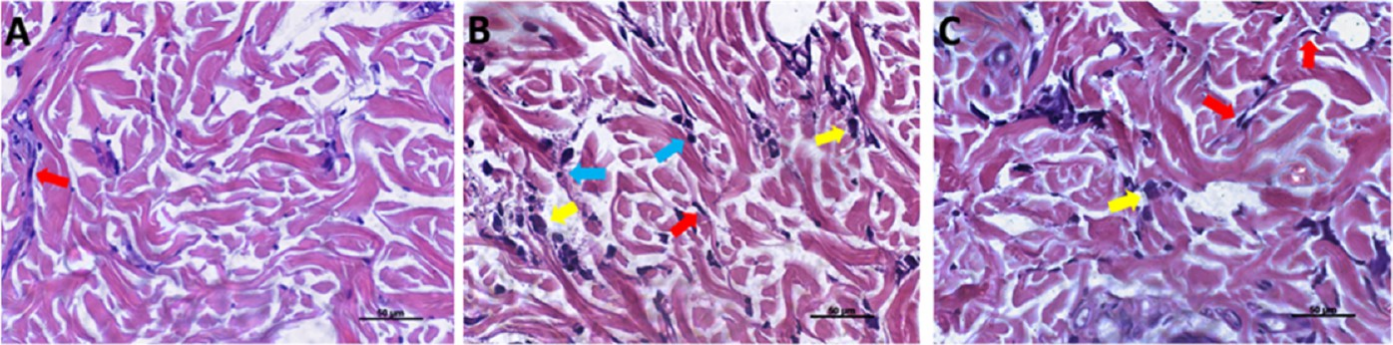
Photomicrographs of the dermal area at 15 days for the PBS group (A), BNp group (B), and AANp group (C). Stained with Hematoxylin and Eosin. In all three groups, dispersed fibroblasts (red arrows) are observed in the collagen matrix, with few inflammatory cells predominantly consisting of macrophages (yellow arrows) and lymphocytes (blue arrows). The density and thickness of collagen fibers in the test groups and the control group.

At 30 days, the regions infiltrated with Nps exhibited a higher density and thickness of collagen fibers throughout the dermal layer. In many specimens, collagen neoformation extended particularly into the basal layer of the dermis, intertwining with the adipose tissue up to the interface between the skin and the underlying muscle. In all groups, the inflammatory infiltrate was mild, and in two specimens from the control group, it was absent. The presence of immature collagen was more evident in groups BNp and AANp compared to the PBS group (Figure 5). In this context, the visualization of collagen in groups AANp supports the findings obtained from the hydroxyproline assay (Figure 5). Interestingly, after the initial robust stimulation promoted by BNp, by day 30, it was also possible to observe young fibers undergoing remodeling, even without a detectable increase in collagen levels assay (Figure 5).

**Figure 5:**
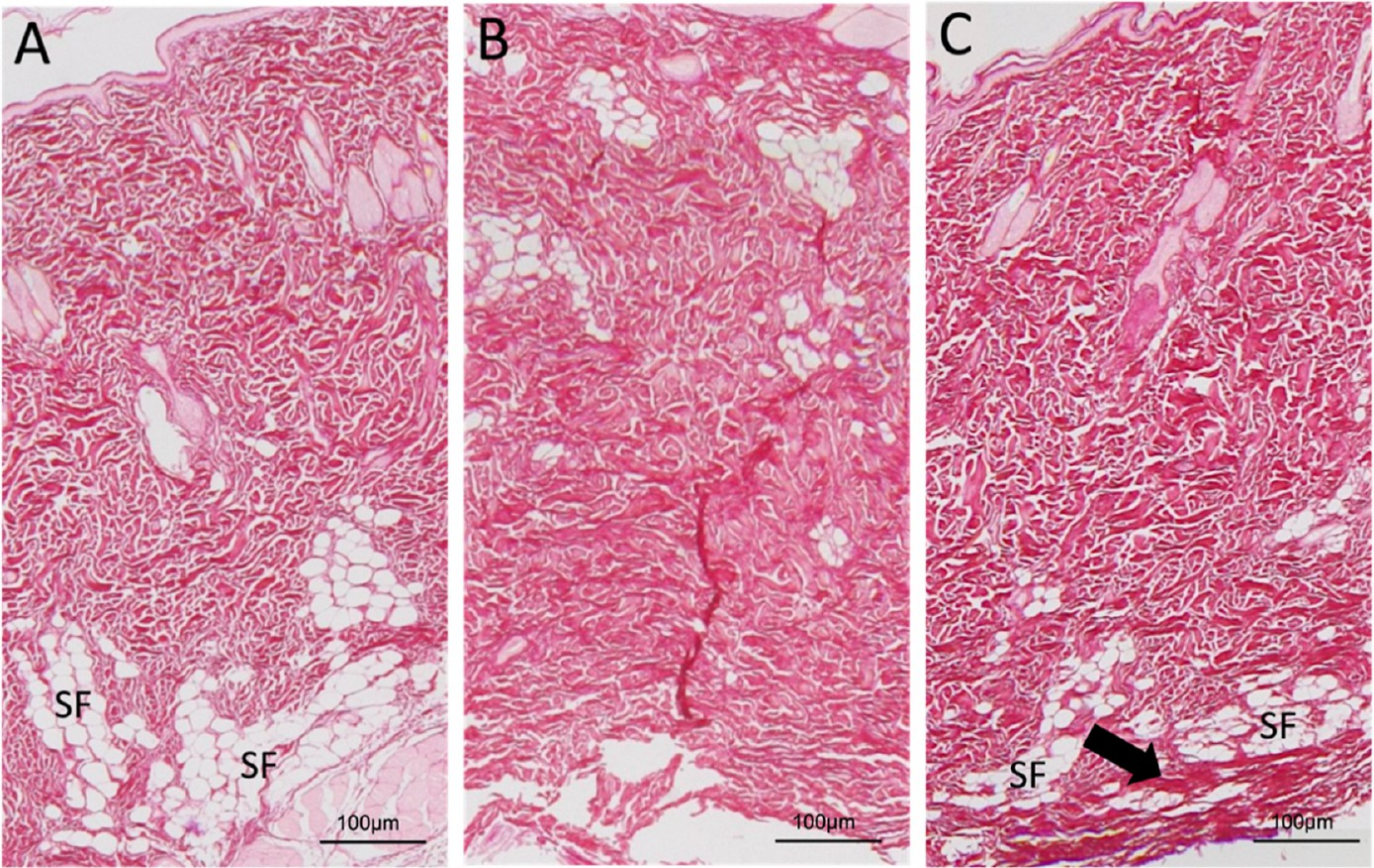
Photomicrographs of the dermal area at 30 days for the PBS group (A), BNp group (B), and AANp group (C). Stained with Picrosirius. Note the increase in density and thickness of collagen fibers in images B and C. The arrow indicates the formation of collagen bundles in the basal portion of the dermis, interspersing with subcutaneous fat up to the boundary of the muscle layer (SF) Subcutaneous fat.

At 60 days, the areas treated with the formulations exhibited denser and thicker collagen bundles compared to the control group, adopting a more parallel arrangement to the dermal surface than those observed in the 15- and 30 day specimens (Figures 6 and 7). Inflammatory cells were infrequent or absent in many samples. Multinucleated giant cells were not observed in and specimen at any of the analysis time points. Furthermore, at 60 days, both groups exposed to Nps showed a predominance of immature collagen, indicating a young matrix undergoing remodeling (Figure 8), despite the collagen levels not showing a detectable increase assay (Figure 5).

**Figure 6:**
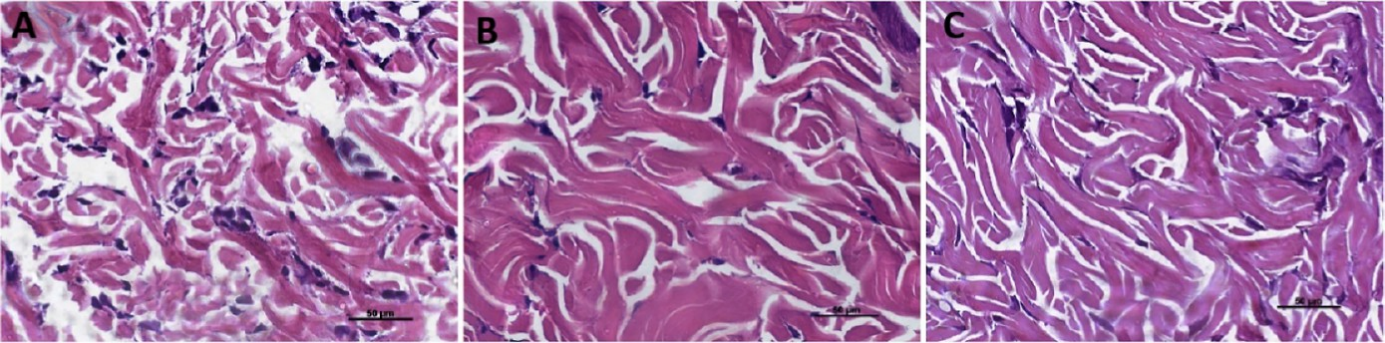
Photomicrographs of the dermis at 60 days for the PBS group (A), empty Np group (B), and NpAA group (C). Stained with Hematoxylin and Eosin. Note the thicker, denser collagen bundles in images B and C, along with the low number of inflammatory cells.

**Figure 7:**
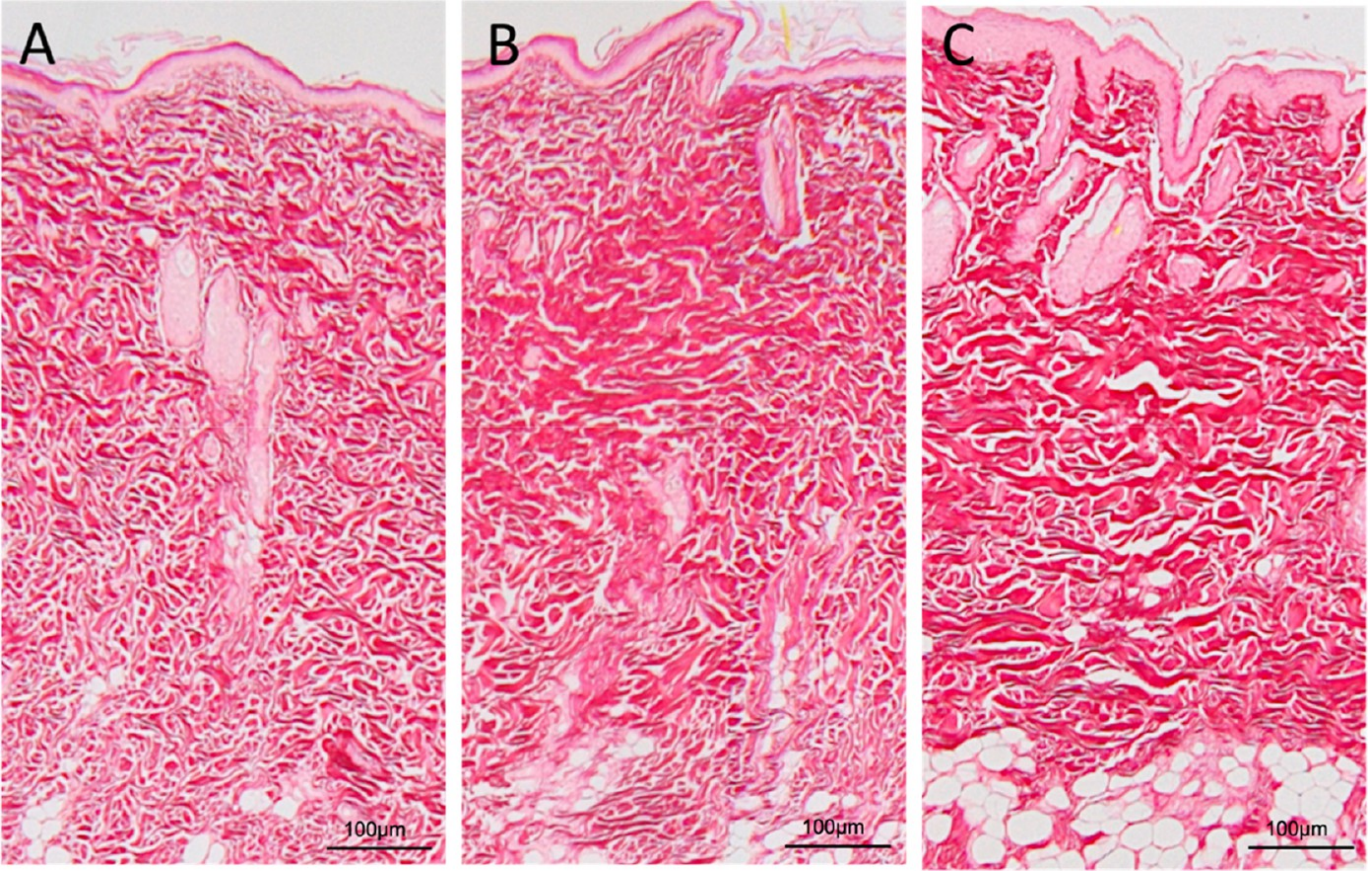
Photomicrographs of the dermal area at 60 days for the PBS group (A), empty Np group (B), and NpAA group (C). Stained with Picrosirius. Increased density and thickness of collagen bundles in B and C compared to the control image (A). The neoformed collagen assumes a more parallel orientation to the skin surface.

**Figure 8:**
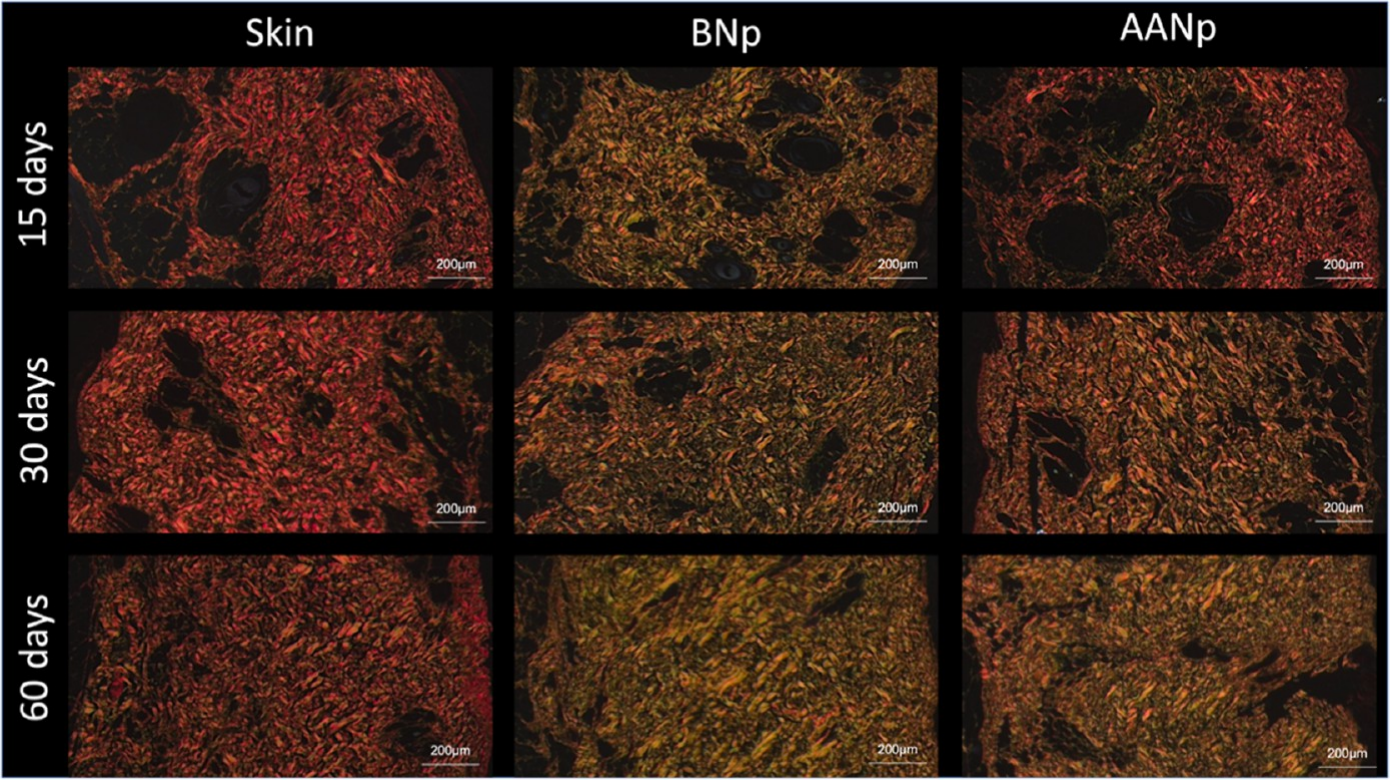
Distribution of collagen fiber maturation in the different analysis groups (Picrosirius, polarized light in dark field). Predominance of orange and greenish fibers in the test groups at 15, 30, and 60 days, indicating a continuous inflammatory process of collagen neogenesis after nanoparticle injection.

By analyzing the density of collagen bundles stimulated by the nanoparticles at 60 days, it was observed that the newly formed collagen showed an increase in thickness and adopted a parallel orientation to the skin surface. Based on the minimal local inflammation and the absence of multinucleated giant cells, along with the nanometric scale of the particles, a higher degree of safety is suggested compared to the nodule formation reported with the use of PLLA.^49^

## Conclusion

The nanoparticles exhibited an average diameter below 300 nm, a moderately monodisperse distribution (PDI <0.2), and spherical morphology. AA was efficiently encapsulated, and all formulations exhibited biocompatibility, with cytotoxicity observed only in macrophages at the highest concentrations of BNp and AANp; no toxicity was detected in epithelial and fibroblast cells. The hydrogel demonstrated good stability and spreadability. The findings demonstrated that nanosystems can be important allies in dermal remodeling (increasing the Hydroxyproline/Collagen production), especially when combining a biocompatible polymer with ascorbic acid. Significant dermal remodeling was observed with the aforementioned combination, yet without a notable presence of inflammatory infiltrate. Thus, it is concluded that nanotechnology may represent a significant advancement as a primary therapy or as a complementary therapy to microparticulate collagen biostimulators, offering reduced risks of complications and a broader range of indications.

## Supplementary Material



## Notes

Suitability parameters for ascorbic acid and hydroxyproline quantification (Figure S1); and *in vitro* cytotoxicity assessment of Poloxamer 188 and ascorbic acid on Vero, J774A.1, and HFF-1 cells (Figure S2) (PDF)

## Abbreviations

%EE: encapsulation efficiency percentage
AA: ascorbic acid
AANp: ascorbic acid-loaded nanoparticles
ANOVA: one-way analysis of variance
BNp: blank nanoparticles
CEUA: animal ethics committee
CH_3_COOH: acetic acid
Cl_3_FeH_12_O_6_: iron­(III) chloride hexahydrate
CMC: carboxymethylcellulose
CO_2_: carbon dioxide
CuSO_4_: copper­(II) sulfate
DLS: dynamic light scattering
DMEM: Dulbecco’s modified eagle’s medium
DMSO: dimethyl sulfoxide
EDTA: ethylenediaminetetraacetic acid
Ei: spreadability
FBS: fetal bovine serum
FDA: food and drug administration
H&E: hematoxylin & eosin
HFF-1: human foreskin fibroblast cell line
IC: slope of the linearity curve
LOD: limit of detection
LOQ: limit of quantification
MTT: (3-(4,5-dimethylthiazol-2-yl)-2,5-diphenyltetrazolium bromide) cytotoxicity assay
Np: nanoparticles
PDI: polydispersity index
PBS: phosphate-buffered saline
Phen: 1,10-phenanthroline
pH: hydrogen ion concentration
PLGA: poly­(lactic-*co*-glycolic acid)
PLLA: poly-l-lactic acid
PMMA: poly­(methyl methacrylate)
ROI: region of interest
SD: standard deviation
TEM: transmission electron microscopy
UVA: ultraviolet A radiation
UVB: ultraviolet B radiation
ζ (Zeta): zeta potential
