# Supplementary material for: In Vivo Biostimulatory Efficacy of Ascorbic Acid-Loaded Poly(lactic-co-glycolic Acid) Nanoparticles Hydrogel for Dermal Remodeling
Source: ACS Omega. 2025 Sep 11;10(37):42300–12. doi: 10.1021/acsomega.5c01910 (PMC12461425; doi:10.1021/acsomega.5c01910)
Supplement: Supplementary file 1 [file ao5c01910_si_001.pdf]

In vivo bio-stimulatory efficacy of ascorbic acid-loaded poly(lactic-co-glycolic acid) nanoparticles hydrogel for dermal remodeling

*Anna Raphaella Autran Colaço[a], Priscila de Souza Furtado[b], Daniel Figueiredo Vanzan[b], Nicole Serqueira da Silva[c], Flávia Almada do Carmo[b], Lucio Mendes Cabral[b], Alice Simon[b], Jônatas Caldeira Esteves[c], Plínio Cunha Sathler[a]**

[a]Universidade Federal do Rio de Janeiro, Departamento de Análises Clínicas e Toxicológicas, Faculdade de Farmácia, Ilha do Fundão, CEP 21941-902, Rio de Janeiro, RJ, Brazil.

[b]Universidade Federal do Rio de Janeiro, Departamento de Fármacos e Medicamentos, Faculdade de Farmácia, Ilha do Fundão, CEP 21941-902, Rio de Janeiro, RJ, Brazil.

[c]Universidade Federal do Rio de Janeiro, Departamento de Clínica Odontológica, Faculdade de Odontologia, Ilha do Fundão, CEP 21941-902, Rio de Janeiro, RJ, Brazil.

Supporting Information

Quantification	Suitabiliity parameters		Results
**Ascorbic acid**	Linearity (1-40 µg/mL)	Slope	0.0030 ± 0.0004
		Intercept	0.0452 ± 0.0037
		Correlation coeficiente (r)	0.9979 ± 0.0013
	Limit of Detection		4.07 µg/mL
	Limit of Quantification		12.34 µg/mL
**Hydroxyproline**	Linearity (12.5-200 µg/mL)	Slope	0.0111 ± 0.0008
		Intercept	0.125 ± 0.011
		Correlation coefficient (r)	0.9982 ± 0.00052
	Limit of Detection		3.27 µg/mL
	Limit of Quantification		9.91 µg/mL

Figure S1. Results of the suitability parameter established for ascorbic acid and hydroxyproline quantification. Data expressed as mean ± standard deviation (SD); n=3

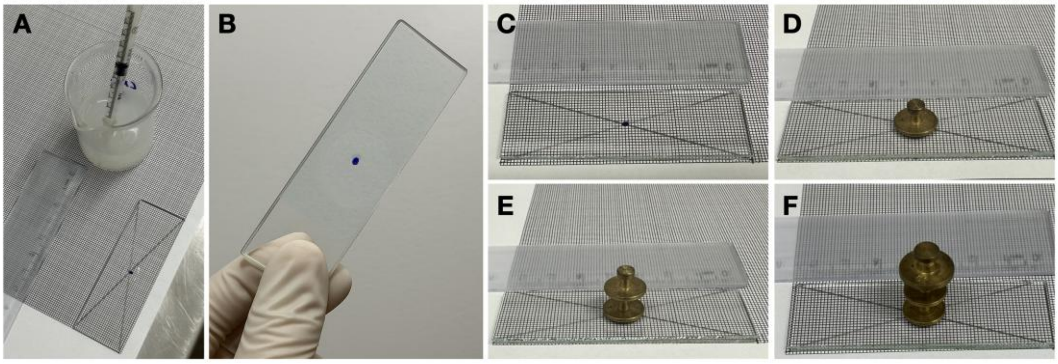

Figure S2. Visual appearance of the gel formulation (A–B). Spreadability test procedure (C–F): (C) placement of a pre-weighed glass slide over the sample; (D) addition of a 2 g weight; (E) addition of a second 2 g weight; (F) addition of the final 5 g weight.

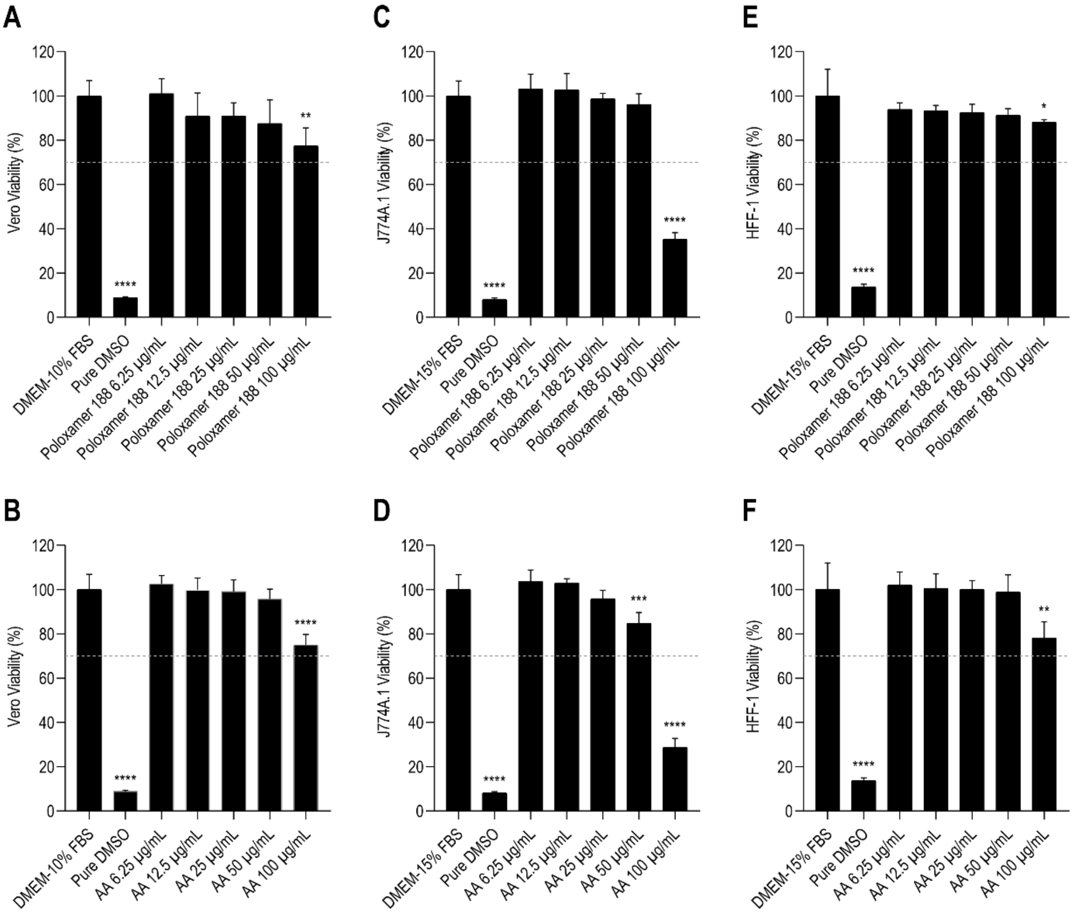

Figure S3. *In vitro* assessment of the cytotoxic effect of Poloxamer 188 and AA on Vero (A and B), J774A.1 (C and D), and HFF-1 (E and F) cells at concentrations ranging from 6.25 to 100 µg/mL. DMEM-10% FBS and DMEM-15% FBS were used as negative controls, and pure DMSO

as the positive control. Results are expressed as mean ± standard deviation (SD); n=4. (* $p < 0.05$; ** $p < 0.005$; *** $p < 0.0005$; **** $p < 0.0001$).
